# The complete mitochondrial genome of the plumed worm *Diopatra cuprea* (Annelida: Onuphidae)

**DOI:** 10.1080/23802359.2021.2008838

**Published:** 2021-12-10

**Authors:** Lauren E. Hamilton, Gabriel C. Preston, Jade B. Wheeler, Alexis M. Janosik, Viktoria E. Bogantes

**Affiliations:** Department of Biology, University of West Florida, Pensacola, FL, USA

**Keywords:** mtDNA, Gulf of Mexico, phylogenetics, evolution

## Abstract

In this study, we describe the complete mitochondrial genome of *Diopatra cuprea* (Bosc, 1802). The mitogenome was found to contain 14,990 base pairs (67.53% A + T content), with a total of 37 genes (13 protein coding, 22 transfer RNAs, and 2 ribosomal RNAs). This study also examined mitogenome phylogenetics relationships of closely related species and recovered that *D. cuprea* is closely related to eunicids. This work has added to the genetic resources for furthering evolutionary studies of Annelida.

The species *Diopatra cuprea* (Bosc, 1802), commonly known as the plumed worm, is an annelid worm in the family Onuphidae. *Diopatra cuprea* builds capped, cylindrical spiral tubes made from debris, such as shells, sediment, pebbles, and plant matter (Konhauser et al. 2020). The constructed tube provides protection and facilitates food gathering for the worm. Stable isotope analyses suggest that members of Onuphidae are broad omnivores, using jaws to capture organic material for consumption (Pardo and Amaral 2006; Jumars et al. 2015). Along with other polychaetes, *D. cuprea*, is important to the ecosystem not only as prey, but also in terms of bioturbation (Woodin 1974), ensuring mixing of organic material in the sediment surrounding the tube (Matsui et al. 2004). Geographically, *D. cuprea* can commonly be found along the western Atlantic coast, in the Caribbean Sea, and in the Gulf of Mexico (Konhauser et al. 2020). This taxon is considered a species complex with current taxonomy hindering species diversity; a recent study using morphological and molecular data resulted in the description of four new species previously identified as *D. cupre*a for the Brazilian coast (Seixas et al. 2021). This discovery of previously undescribed species highlights the need for DNA resources. In this study, we described the complete mitochondrial genome of *Diopatra cuprea*, sampled from the panhandle of Florida.

*Diopatra cuprea* was collected from Apalachee Bay, Wakulla county, Florida, United States (30°02′56.8″N, 84°04′48.7″W) and preserved in 200 proof ethanol. The specimen was deposited in the invertebrate collection at Florida Museum of Natural History (www.floridamuseum.ufl.edu, John D. Slapcinsky, slapcin@flmnh.ufl.edu) under voucher number Annelida 009278. Total genomic DNA was extracted using the DNeasy Blood and Tissue kit (Qiagen). DNA libraries were constructed using Illumina HiSeq (Illumina, San Diego, CA), and were sequenced using HiSeq platform, with 250-bp paired-end reads at the Hubbard Center for Genomics, Sequencing Core Facility (Durham, NH). Sequencing resulted in 7,671,076 raw reads, which were trimmed and normalized using Geneious Prime V. 2021.0.3 (https://www.geneious.com), followed by *de novo* assembly. The assembled genome was annotated using MITOS2 (Bernt et al. 2013). To infer phylogenetic placement, a maximum-likelihood phylogenetic tree was constructed using MEGA-X (Kumar et al. 2018) with default settings and 1000 bootstrap replicates (Figure 1). The phylogenetic placement of *D. cuprea* was investigated using six other complete mitochondrial genomes of related species: *Eunoe nodosa* (MW557378.), *Gesiella jameensis* (MV794260.1), *Glycera dibranchiata* (KT989318.1), *Marphysa sanguinea* (KF33802.1), *Marphysa tamurai* (MG205526.1), and *Orbinia laterillii* (AY961084.1). Annotation of the assembled genome was conducted with MITOS2 (Bernt et al. 2013).

**Figure 1.**
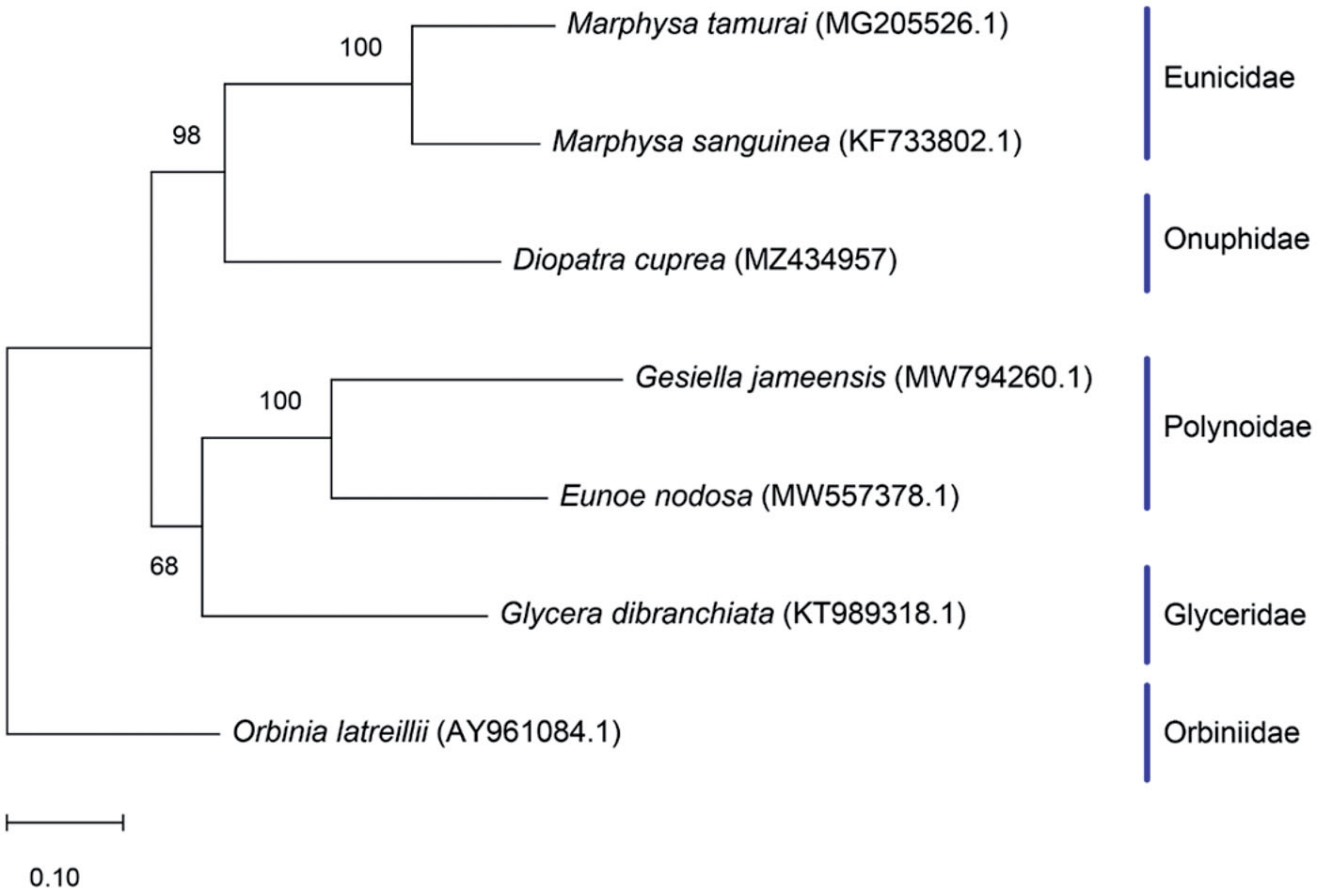
Maximum likelihood tree showing the phylogenetic relationship of *Diopatra cuprea* based on the full mitochondrial genomes of six other worm species, with *Orbinia latreillii* as the outgroup. Bootstrap values are indicated at the nodes.

The complete mitogenome of *Diopatra cuprea* was 14,990 bp in length (GenBank accession number: MZ434957), with an adenine and thymine concentration of 67.53% (A 29.85% and T 37.68%) and guanine concentration of 10.52% and cytosine concentration of 21.95%. The complete mitochondrial genome contained 13 protein-coding-genes, 22 tRNA genes, and 2 ribosomal RNA genes (rrnS and rrnL). The standard start codon is ATG (making up 64% of start codons) and the stop codon is TAA (making up 57% of stop codons). *Diopatra cuprea* clustered with *M. sanguinea* and *M. tamurai*, two members in the Eunicidae family, which is sister to *G. dibranchiata* and two polynoids.

## Data Availability

The data that support the findings are openly available in NCBI (https://www.ncbi.nlm.nih.gov/), reference number (MZ434957). The associated BioProject, SRA, and Bio-Sample numbers are PRJNA731158, SRR14596718, and SAMN19272656 respectively.
